# Analytic framework for peptidomics applied to large-scale neuropeptide identification

**DOI:** 10.1038/ncomms11436

**Published:** 2016-05-04

**Authors:** Anna Secher, Christian D. Kelstrup, Kilian W. Conde-Frieboes, Charles Pyke, Kirsten Raun, Birgitte S. Wulff, Jesper V. Olsen

**Affiliations:** 1 Faculty of Health and Medical Sciences, Novo Nordisk Foundation Center for Protein Research, University of Copenhagen, Blegdamsvej 3b, DK-2200 Copenhagen, Denmark; 2 Histology and Imaging, Novo Nordisk A/S, Novo Nordisk Park, DK-2760 Maaloev, Denmark; 3 Protein & Peptide Chemistry, Novo Nordisk A/S, Novo Nordisk Park, DK-2760 Maaloev, Denmark; 4 Incretin & Obesity Pharmacology, Novo Nordisk A/S, Novo Nordisk Park, DK-2760 Maaloev, Denmark; 5 Incretin & Obesity Research, Novo Nordisk A/S, Novo Nordisk Park, DK-2760 Maaloev, Denmark

## Abstract

Large-scale mass spectrometry-based peptidomics for drug discovery is relatively unexplored because of challenges in peptide degradation and identification following tissue extraction. Here we present a streamlined analytical pipeline for large-scale peptidomics. We developed an optimized sample preparation protocol to achieve fast, reproducible and effective extraction of endogenous peptides from sub-dissected organs such as the brain, while diminishing unspecific protease activity. Each peptidome sample was analysed by high-resolution tandem mass spectrometry and the resulting data set was integrated with publically available databases. We developed and applied an algorithm that reduces the peptide complexity for identification of biologically relevant peptides. The developed pipeline was applied to rat hypothalamus and identifies thousands of neuropeptides and their post-translational modifications, which is combined in a resource format for visualization, qualitative and quantitative analyses.

## Abstract (web summary)

Neuropeptide research is challenged by technical difficulties in identifying new bioactive peptides. Here the authors present an analytical pipeline for large-scale peptidomics applied to the rat hypothalamus, identifying thousands of endogenous neuropeptides and their post-translational modifications.

## Graphical abstract

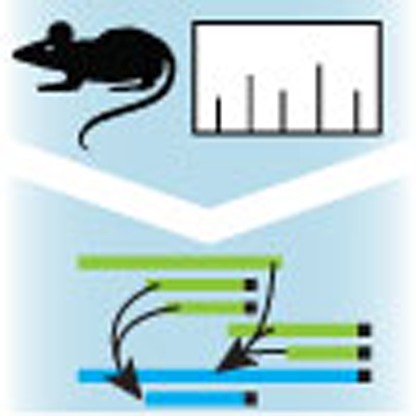

Analogues of bioactive peptides like glucagon-like peptide 1 (GLP-1) are emerging as prominent drugs for treatment of metabolic disorders such as diabetes and obesity. As a consequence of this, the analysis of endogenous peptides from tissues holds a great promise for drug discovery including the identification of new, bioactive peptides. Neuropeptides are peptide hormones in the brain, which elicit key signalling responses that affect diverse behavioural end endocrine functions including weight homeostasis, pain and psychiatric disorders^1^. Neuropeptide research is challenged by difficulties in identifying new bioactive neuropeptides, but the emergence of a new generation of high-performance mass spectrometers (MS) makes large-scale identification of endogenous peptides extracted from tissue samples possible, a strategy referred to as peptidomics^2^,^3^. This enables unbiased and explorative studies and in principle allows for the identification of post-translational modifications (PTMs)^4^,^5^ as well as previously undescribed neuropeptides^6^,^7^. Analysis of peptidomes has so far been challenged by technical issues due to unspecific protease digestion during sample preparation and computational challenges in data analysis as well as difficulties in the biological interpretation. This calls for development of new sample preparation methods and bioinformatic approaches to reliably identify new potential neuropeptides^8^. Previous studies show that heat inactivation either performed by focused microwave irradiation^9^,^10^,^11^, by heating the excised tissue in a conventional microwave oven^12^ or by specialized controlled-heating instruments^13^ largely prevents the production of proteolytic peptide fragments when compared with traditional protocols based on snap freezing^12^,^13^. Furthermore, several strategies have been reported for identification of neuropeptides from complex and large data sets based on cleavage analysis and *de novo* sequencing^14^,^15^.

Here, we describe a compilation of methods into a simple and robust analytic framework for extracting, analysing and identifying endogenous peptides in rat brain. Different heat inactivation procedures were compared and combined with protease inhibitor perfusion of animals, to further retain intact peptides in the sample. The peptidomes extracts were analysed by single-shot nanoflow liquid chromatography in line with high-resolution tandem mass spectrometry. A sequential, computational framework was developed to efficiently analyse the resulting large data set in a stringent approach minimizing errors of false peptide identifications. As proof-of-concept, the methodology was applied to large-scale neuropeptide identification from rat hypothalamus resulting in thousands of identified neuropeptides. In addition, an abundance of PTMs on these peptides are identified, and these data are combined in a resource format for visualization, qualitative and quantitative analyses.

## Results

### Benchmarking of optimized peptidomics workflow

**Figure 1:**
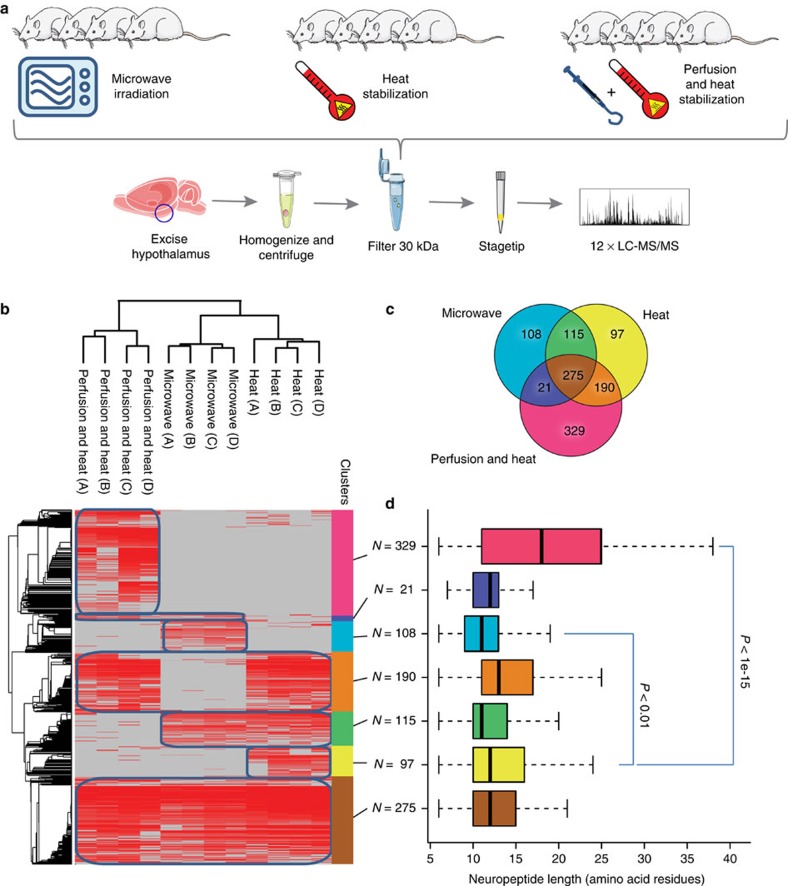
Comparison of peptidomics sample preparation methods. (**a**) Workflow using microwave-irradiation, heat-stabilization or protease inhibitor perfusion before heat stabilization. Endogenous peptides from rat hypothalamus were extracted by homogenization in an urea-buffer and filtration using a 30-kDa cutoff filter. Peptides were desalted and concentrated on C_18_-STAGE tips and analysed by LC-MS/MS. (**b**) Hierarchical clustering of identified neuropeptides. (**c**) Venn diagram of identified neuropeptides. (**d**) Analysis of neuropeptide length distributions. Box-plot analysis of neuropeptide length represented as number of amino-acid residues. Illustrations were generated using images from Servier.com.

**Figure 2:**
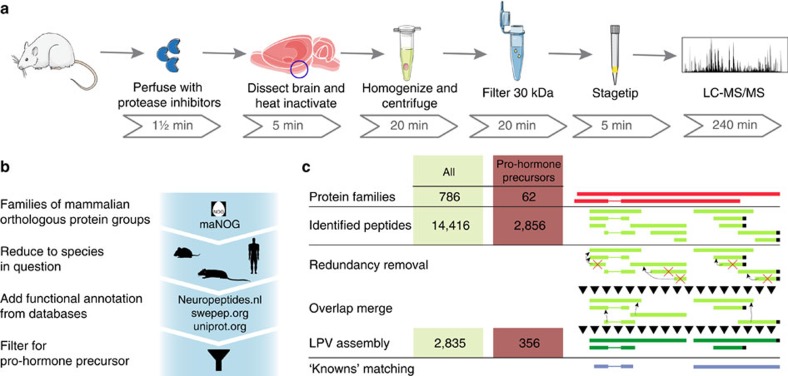
Analytical framework for analysis of endogenous peptides. (**a**) Optimized sample preparation protocol for experimental isolation of endogenous peptides from the hypothalamus before mass spectrometric analysis. (**b**) Computational data analysis was done in the context of orthologous protein groups enabling comparison between species and incorporation of previously annotated peptides from multiple databases and online resources to focus the analysis on a subset of peptides. (**c**) Generation of LPVs is schematically shown as removal of redundancy and merging of overlapping peptides. Illustrations were generated using images from Servier.com.

To establish the optimal sample preparation method for mass spectrometry-based peptidomics, different published methods for neuropeptide extraction from the tissue^12^,^13^,^16^ were compared in terms of sample recovery and proteolytic breakdown products. A simple denaturing buffer consisting of 8 M urea was utilized as extraction media based on previous publications^16^, and neuropeptides were enriched from larger protein fragments by centrifugation through 30 kDa cutoff filters before analysis by liquid chromatography tandem mass spectrometry (LC-MS/MS). To allow further sub-dissection of complex biological tissues such as hypothalamus without rapid peptide degradation, heat stabilization by microwaving the excised tissue immediately following decapitation^12^ was compared with controlled conductive heating, to generate rapid, homogenous, thermal denaturation of the dissected brain using a commercially available instrument^13^ and finally in combination with perfusion with protease inhibitors (Fig. 1a). Each of the three protocols were performed as biological quadruplicates (*N*=4) and analysed by 2 h LC-MS/MS runs using high-resolution orbitrap tandem mass spectrometry. The complete data set of 12 raw files were analysed together using the MaxQuant software suite (http:// www.maxquant.org), which resulted in the identification of 11,509 modification-specific peptide variants. To assess the reproducibility, a hierarchical clustering of the 1,135 unique neuropeptides mapping to annotated pro-hormone protein precursors and identified in at least 2 of 12 samples were performed and the samples clustered according to sample preparation method (Fig. 1b). To quantify neuropeptide recovery, each cluster was visualized in a Venn diagram (Fig. 1c). When comparing number of peptides from the two heat inactivation methods, the commercial heat stabilization generated a higher number of peptides. To further restrict post-mortem proteolytic degradation, perfusion with a protease inhibitor cocktail combined with heat inactivation of the tissue was applied. Addition of this step increased the length of retrieved peptides significantly more than heat stabilization by itself (Fig. 1d). This optimized sample preparation protocol for rapid peptide extraction is schematized and can be performed within an hour (Fig. 2a).

### Large-scale peptidomics applied to hypothalamus

To apply this protocol to large-scale peptidomics, 32 rats were perfused with protease inhibitors and their brains heat inactivated. Endogenous peptides were extracted in parallel from the stabilized hypothalamic tissue by sonication and larger protein fragments removed by molecular weight cutoff spin filters. The resulting peptidomes were analysed by LC-MS/MS resulting in identification of 16,037 unique modification-specific peptide variants covering 14,416 unique peptide sequences (Supplementary Data 1). Biological replica had an overlap based on unique peptide sequence of 68±4% (±standard deviation). Including the MaxQuant software feature ‘match between runs', an average overlap of 78±5% was observed. The high data quality is evidenced by the technical details including low mass errors, high peptide scores and reproducibility (Supplementary Fig. 1).

A large fraction of the peptides were derived from intracellular proteins likely originating from tissue damage and did not constitute neuropeptide candidates. To differentiate between these, the list of peptides was organized by membership to protein families according to an existing mammalian orthologous group framework^17^. This enabled a general high level aggregation of information from publically available protein databases (Uniprot.org, Swepep^18^ and Neuropeptides.nl) across several species (Fig. 2b) and provided a curated database of 182 protein families containing pro-hormone precursors, ∼1% of a total 18,972 orthologous protein groups. Applied to our data, the identified peptides mapped to 786 orthologous protein groups of which 62 belonged to the pro-hormone precursors (Supplementary Data 1).

### Analysis of longest peptide variants (LPVs)

To reduce the complexity of our data set further, we developed an algorithm that assembled the peptides into LPVs (Fig. 2c). As nonspecific protease activity generates ladder series of peptides with a single amino acid removed, a bioinformatic merge of overlapping sequences enabled a focus on more specific protease activity. This reduced the 14,416 unique peptides to 2,835 LPVs of which specifically 356 LPVs belonged to the pro-hormone precursor group (Supplementary Data 2). These overlapped with 251 previously annotated neuropeptides of which 45 were found to be identical full-length peptide matches including neuropeptide-Y and galanin. Furthermore, 105 previously undescribed LPVs were derived from pro-hormone precursors, but without known biological activity. To condense information, the full peptide list was compiled into a database and visualized as presented (Supplementary Data 3). Essentially all neuropeptides previously described to be present in the hypothalamus were identified including agouti-related peptide, which is only expressed in a very small part of the hypothalamus termed the arcuate nucleus^19^. Several other well-described neuropeptides were identified including glucagon-like peptide-1, α-melanocyte-stimulating hormone (α-MSH) and somatostatin, as well as bioactive peptides derived from chromogranins, cholecystokinin, secretogranins and cocaine- and amphetamine-regulated transcript. The full peptide list can be found in Supplementary Data 2.

### Comparison with other bioinformatic tools

To benchmark our bioinformatic workflow described above, we compared it with established approaches such as a curated database of neuropeptides (for example, NeuroPep^20^) and prediction tools (for example, PeptideRanker^21^ or NeuroPred^22^). A direct comparison of our peptide data to the NeuroPep database showed that of the 2,856 unique sequences mapping to pro-hormone precursors, 2,015 sequences were deemed neuropeptides by both approaches and only 34 peptides belonging to Tubulin beta-3 and Nucleobindin-1 were unique to the NeuroPep database, but were not determined as pro-hormone precursors in our workflow. Conversely, 841 neuropeptides were only found by our approach originating from more than 30 protein families (Supplementary Fig. 2a). Next, we analysed our peptidomics data set of 14,416 identified peptides with PeptideRanker, which is a peptide-level predictor for bioactivity. A minimal difference in predicted bioactivity was found between the 2,856 peptides from the pro-hormone precursor group and the rest (Supplementary Fig. 2b). However, LPVs derived from pro-hormone precursors showed an overall increase in their predicted bioactivity probability both compared with all peptides and to the other LPVs that achieved significantly lower bioactivity probabilities. Similar results were observed when comparing to the protease prediction tool NeuroPred. In this analysis, only 3.6% (103/2,856) of the cleavage sites overlapped between predicted and observed peptides originating from pro-hormone precursors. However, the LPVs from the pro-hormone precursor group showed a significantly larger overlap of 15.7% (56/356).

### Gene ontology analysis of peptidomics data

**Figure 3:**
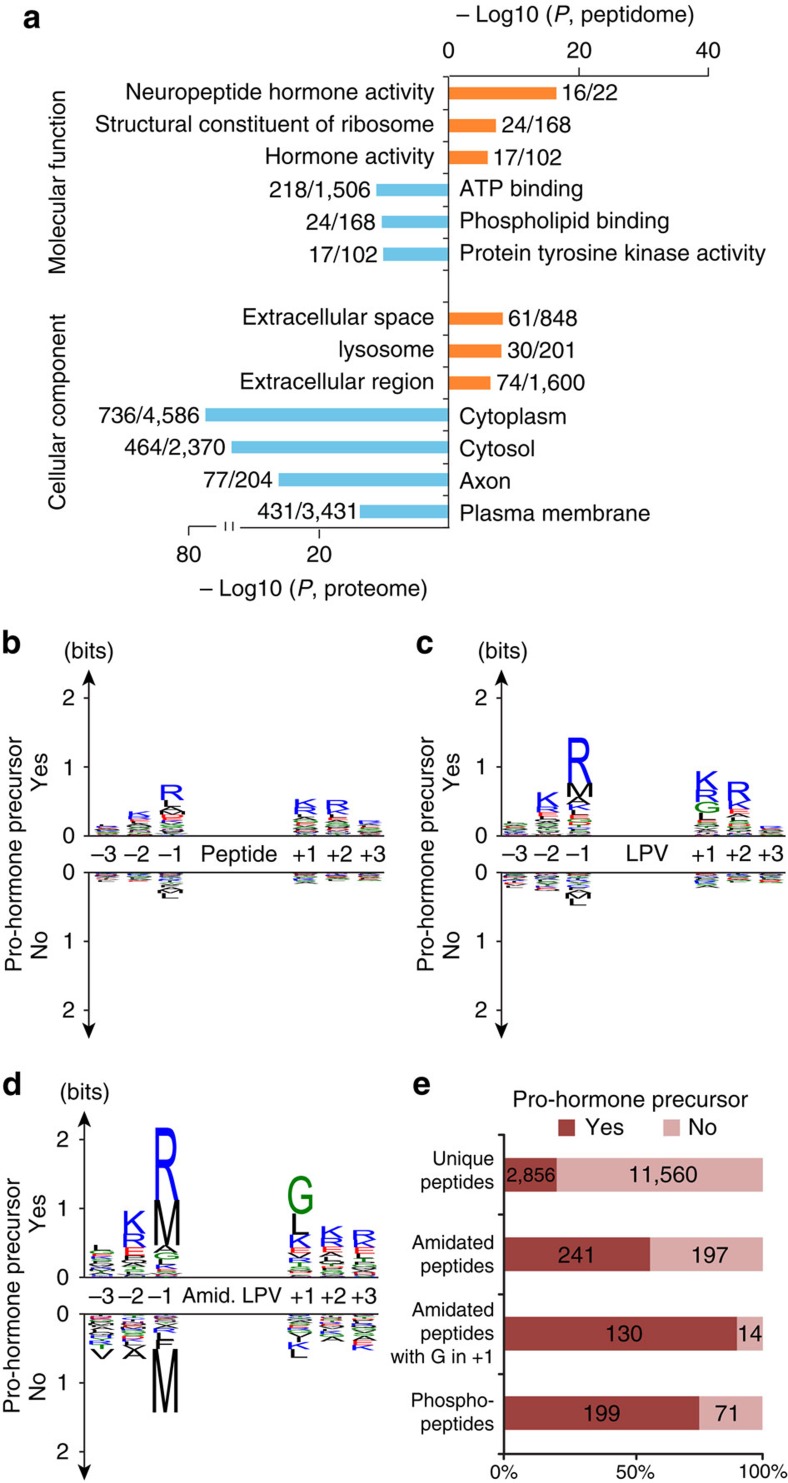
Neuropeptide feature analysis. (**a**) Gene ontology enrichment comparing the identified peptidome to its corresponding hypothalamic proteome. (**b**) Logo plots for the C- and N-terminal regions flanking peptides split by pro-hormone precursor group membership. (**c**) Logo plots for the C- and N-terminal regions flanking LPVs split by pro-hormone precursor group membership. (**d**) Logo plots for the C- and N-terminal regions flanking amidated LPVs split by pro-hormone precursor group membership. (**e**) Overview of number of identifications and their modification state. In total, 14,416 unique peptides sequences were found, 20% were found in orthologous protein groups that contained a pro-hormone precursor.

To evaluate the efficiency of neuropeptide recovery and to examine the functional profile of all the identified peptide sequences, a gene ontology (GO) enrichment analysis was performed, where the cellular localization and molecular function of the identified endogenous peptidome was compared with the hypothalamus proteome derived by mass spectrometric analysis of a tryptic digest of the proteins retained in the spin filter (Fig. 3a). The most significantly enriched molecular function classifier in the peptidome was neuropeptide hormone activity, whereas the most underrepresented classifier was ATP binding, typically representing intra-cellular proteins. For GO classifiers of cellular localization, the most enriched in the peptidome was extracellular space, whereas the cytoplasm was most enriched in the reference proteome. The results conformed well to the known function and cellular organization of neuropeptides and underscored that our protocol effectively separated neuropeptides from other polypeptides.

### Analysis of protease cleavage preferences

A linear sequence motif analysis revealed that the most prevalent amino acids preceding the N-termini of the peptides from the pro-hormone precursor group were dibasic cleavage sites KR or monobasic site R (Fig. 3b). This overrepresentation of basic amino acids preceding the N-termini was even stronger for the 356 LPVs derived from the pro-hormone precursor group (Fig. 3c). The most abundant residues trailing the C-termini were KR in non-amidated neuropeptides and a C-terminal glycine in amidated neuropeptides followed by K/R, in line with previous reports^14^,^23^. C-terminal amidation of endogenous peptides such as neuropeptides is often essential for full biological activity. It is well-established that the bifunctional enzyme peptidyl-glycine alpha-amidating monooxygenase, which is abundantly present in our samples, has the ability to convert peptides that terminate in glycine to the corresponding des-glycine peptide amide^24^. These motifs were significantly overrepresented compared with the remaining LPVs or all peptides (Fig. 3d) validating our grouping approach when compared with prior knowledge of pro-hormone precursors.

### Functional analysis of α-MSH phosphorylation

To assign potential biological function to previously undescribed peptides, we filtered the data based on C-terminal amidation, which is a well-described PTM in bioactive neuropeptides^25^. A full list of potential, biologically active neuropeptides were generated from the LPVs with criteria based on K/R in position -1 preceding the N-terminus and K/R in position +1 and +2 trailing the C-terminus or C-terminal amidation followed by G in position +1 (Table 1 and Supplementary Data 4).

**Figure 4:**
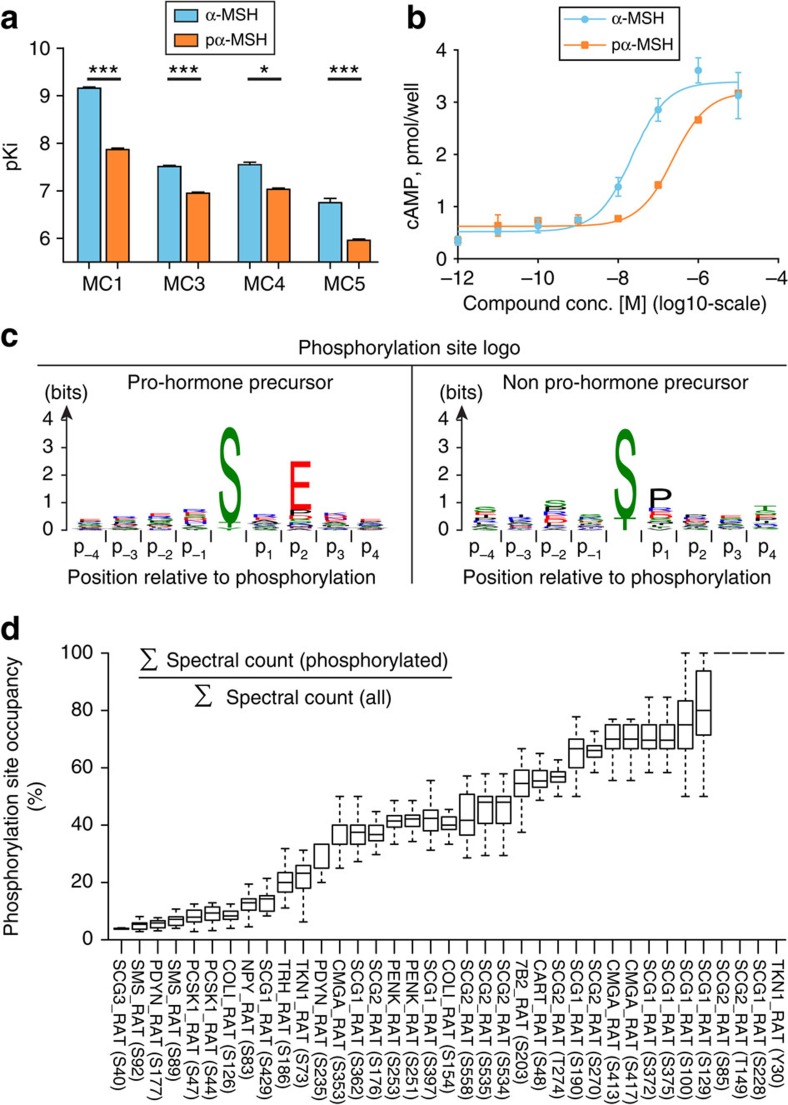
Functional analysis of neuropeptide phosphorylation sites. (**a**) Ki (shown as negative log, pKi) calculated from IC50 values determined under equilibrium conditions in competition with ^125^I-NDP-α-MSH on MC1, 3, 4 and 5 receptors, respectively. (Cheng–Prusoff equation *P<0.05, ***P<0.001). Values are mean±s.e.m. (**b**) Representative figure of α-MSH and phosphorylated α-MSH-stimulated cAMP production in intact BHK cells expressing the human MC4 receptor. (**c**) Logo plots of the phosphorylation sites revealed a [ST]xE motif for the neuropeptide protein precursor group. In contrast, a [ST]P motif was found for the remaining phosphopeptides. (**d**) Occupancy of phosphorylation sites that was observed at least three times. Boxplots depicts variation observed across 32 replicates. Each line contains the Uniprot gene name and phosphorylated position in parentheses.

Besides the 5-fold enrichment in amidated C-termini on neuropeptides, we found an 11-fold over-representation of phosphorylated peptides among the pro-hormone precursor groups (Fig. 3e and Supplementary Data 5). A total of 438 peptides were found to be amidated, of which 55% were found in the neuropeptide pro-hormone precursor group. For phosphorylated peptides, this fraction was 74% out of a total of 270 phosphopeptides. A search against the Uniprot database identified approximately half of the PTMs to be previously undescribed. These modified peptide sequences could potentially represent biologically active peptides or fragments thereof, making them interesting targets to explore further. One of the new PTM identifications was a phosphorylation of the second serine [SYS(ph)MEHFRWGKPV-amide] in the α-MSH peptide corresponding to Serine-126 in the full-length POMC pro-hormone precursor protein. To reveal the functional role of this phosphorylation site, we evaluated whether the phosphorylation altered the *in vitro* activity of the melanocortin receptors (MC1, 3, 4 and 5) targeted by α-MSH as described in ref. 26. The pKi values were calculated from IC50 values determined in radio ligand displacement filtration binding assays to membranes from recombinant BHK570 cells expressing the relevant human melanocortin receptor and using ^125^I-NDP-α-MSH as radio ligand. Phosphorylation of α-MSH lowered its binding affinity by 11-fold for MC4 and 7- to 8-folds for MC5 and MC1 receptors compared with dephosphorylated form of α-MSH (Fig. 4a). Concurrently, the phosphorylation lowered the MC4 activated cyclic AMP (cAMP) response by tenfold (Fig. 4b).

### Kinase-substrate motif analysis

Sequence motif analysis of the phosphorylation sites revealed a significant S-x-E motif for the neuropeptide group, including the functional site in α-MSH, whereas an S/T-P motif was found for the remaining phosphopeptides (Fig. 4c). The neuropeptide phosphorylation motif matched perfectly the substrate specificity of the recently described Fam20c protein kinase that phosphorylates secretory pathway proteins within S-x-E motif^27^. Interestingly, this kinase is also responsible for phosphorylation of the majority of peptides residing within the central nervous system^28^,^29^,^30^. This validated our grouping into secretory and intra-cellular protein families.

### Determination of fractional phosphorylation site stoichiometry

To further elucidate the biological function and abundance of phosphorylation in neuropeptides, the occupancy or fractional stoichiometry of all identified phosphorylation sites was estimated under the assumption that phosphorylation does not significantly change the ionization efficiency of the peptide. Phosphorylation site stoichiometry was estimated by comparing spectral counts of phosphopeptides with their dephosphorylated counterparts for each phosphorylation site that was observed at least three times across the 32 biological replicates (Fig. 4d). This was a reproducible measure across samples and revealed that a majority of phosphorylation sites were of high occupancy of 40–80%, whereas approximately one-third was observed with occupancy below 40%.

## Discussion

In this study, we present a compilation of methods into a swift sample protocol for extraction of neuropeptides, followed by mass spectrometric identification and bioinformatic analysis to tease out potential biologically active neuropeptides and novel PTMs. To our knowledge, this is the first comprehensive peptidomics data set that provides a large number of (full-length) neuropeptides from one extraction analysed by single LC-MS/MS analysis. The developed sample preparation protocol is reproducible, minimizes unspecific post-mortem protease digestion issues and can be performed in less than 1 h from tissue dissection to mass spectrometric analysis.

The initial peptide identification process relies on classical shotgun sequencing by data-dependent acquisition and matching of resulting tandem mass spectra against a full proteome database. This has the benefit of not being tissue or species specific while controlling identification error rates. Compared with using a specialized database such as NeuroPep, our workflow that relies on functional filtering of protein families enables the identification of new interesting peptides. A general trend in comparison between our data set and standard prediction algorithms is an overall low overlap. Based on this, sequence information alone seems to be insufficient to estimate bioactivity or cleavage sites. However, collapsing to LPVs improve the overlap for the positive pro-hormone precursor group, in good agreement with the goal to minimize nonspecific peptidase effects on the data. This indicates that prediction tools provide valuable information when used in combination with the workflow presented here.

The developed and implemented algorithm reduces the peptide complexity and derives LPVs for identification and prioritization of hundreds of biologically relevant peptides. The analytical strategy and way of presenting the peptidomics data in Supplementary Data 1, 2, 3, 4, 5 enable multiple views, combined in a resource format for visualization, qualitative and quantitative analysis, as well as hotlist prioritization.

Even though it has been reported that neuropeptide extraction in urea eliminates some peptides such as α-MSH and β-endorphin^16^, both of these peptides were successfully recovered in full length and identified with the presented sample protocol. Heat inactivation was an efficient method for retrieval of an abundance of neuropeptides but combining this method with perfusion using a cocktail of protease inhibitors increased the recovery by threefold compared with either of the heat-inactivating methods by itself. Furthermore, this compilation of methods greatly increased the length of retrieved peptides, and demonstrated that perfusion with protease inhibitors is a powerful strategy for reducing ‘post mortem' proteolytic breakdown products.

The peptidomics experiments reproducibly identified thousands of endogenous neuropeptides originating from pro-hormone protein precursors covering essentially all known hypothalamic peptides and their PTMs including both widespread serine phosphorylation and C-terminal amidation. More than one hundred phosphorylation sites were identified, of which more than half have not been described before. This included a new site on α-MSH, a neuropeptide with a prominent role in appetite regulation. To test the function of this phosphorylation, we performed different cell-based assays and found that the phosphorylation reduced the affinity of α-MSH for its cognate receptors MC1, 3, 4 and 5 by more than tenfold. MC4 receptors are highly expressed in the brain and MC4 agonists such as α-MSH decrease food intake^31^. Thus, as the identified phosphorylation of α-MSH seems to function through a lowering of MC4 receptor interaction, it may play a role in appetite regulation.

Phosphorylation sites with high occupancy is a general phenomenon observed in specific functional cell states such as mitosis^32^ and it is in general a very good indication that the site may be functional^33^. Our data set pinpoints serine phosphorylation as much more abundant on neuropeptides compared with intracellular proteins and that the identified sites are generally of high stoichiometry and conform to the S-x-E motif likely due to the action of a single secreted kinase Fam20C.

Looking forward, the methodological and computational framework we have developed will be applicable to a number of unsolved questions in biology, for example, to further elucidate the profile of secreted peptides in several tissues or to identify the regulated gastric peptidome following either pharmacological intervention of diabetes treatment or gastric bypass, thus taking discovery of new biology and new treatment methods a large step forward.

## Methods

### Sample preparation

*Rat tissues*. The study was carried out following approved national regulations in Denmark and with animal experimental license granted by the Animal Experiments Inspectorate, Ministry of Justice, Denmark. Tissue treatment protocols were compared by either microwaving the whole heads^12^ or by heat inactivating the brains following dissection^13^. Sprague–Dawley rats (Crl:SD, male, 200 g, Charles River, Germany) were decapitated, and the heads immediately placed in a conventional microwave oven, dorsal side facing downwards and heated at 800 W for 9 s followed by dissection of the brain (Gr 1). In comparison, four SD rats were decapitated and the brains were quickly dissected and heated to 95 °C in an air-evacuated cartridge (Denator T1 Heat Stabilizor, Denator AB; Gr 2). A last set of four SD rats (Gr 3) were anaesthetized with isoflurane and perfused (1½ min, 30 ml min^−1^) with isotonic saline containing protease inhibitors (0.120 mM EDTA, 14 μM aprotinin, 0.3 nM valine-pyrrolidide (custom made) and Roche Complete Protease Inhibitor tablets (Roche), pH=7.4) before being decapitated. The brains were quickly dissected and heated to 95 °C in the same air-evacuated cartridge as described above. Following this, hypothalami from all groups were sub-dissected and kept at −80 °C until further use. In the main study, 32 rats were perfused with protease inhibitors and the brains heat inactivated through the same procedure as described for group 3 above.

### Peptide extraction

Tissue was sonicated on ice (3 × 8 s.) in 8 M urea (5 μl mg^−1^ tissue) and centrifuged at 20,000*g*, 20 min, 4 °C. Supernatant was spun through Microcon YM-30 cutoff filters (Millipore; 20 min, 15,000*g* at 4 °C) pre-rinsed with 2 × 80 μl 20% MeCN/30% MeOH (2 × 15 min., 13,000*g*). The peptides were loaded onto in-house packed reversed-phase C_18_ STAGE tips with two Empore C18 discs preconditioned with 20 μl MeOH, 20 μl 80% MeCN, 0.5% AcOH, 2 × 20 μl 1% trifluoroacetic acid (TFA), 3% MeCN. Stage tips were washed with 2 × 20 μl 8% MeCN, 0.5% AcOH and 1 × 50 μl 0.5% AcOH.

### LC-MS/MS, mass spectrometric analysis

Peptides were eluted into 96-well microtitre plates with 20 μl 40% MeCN, 0.5% AcOH followed by 20 μl 50% MeCN, 0.5% AcOH. Organic solvents were removed by vacuum centrifugation in a speed-vac and dried to ∼2 μl. The peptides were reconstituted with 10 μl of 2% MeCN, 0.5% AcOH, 0.1% TFA. Five microlitres of this eluate was analysed by online reversed-phase C18 nanoscale LC-MS/MS on an LTQ-Orbitrap Velos mass spectrometer (Thermo Electron) using a top10 higher-energy collisional dissociation (HCD) fragmentation method as described previously^34^. The LC-MS analysis was performed with a nanoflow Easy –nLC system (Proxeon Biosystems) connected through a nano-electrospray ion source to the MS. Peptides were separated by a linear MeCN gradient for 220 min in a 15-cm fused-silica emitter in house packed with reversed-phase ReproSil-Pur C18-AQ 3 μm resin (Dr Maisch GmbH). Full-scan MS spectra were acquired from 350 to 1,750 *m*/*z* at a target value of 1e6 and a resolution of 30,000, and the HCD-MS/MS spectra were recorded at a target value of 5e4 and with resolution of 7,500 using a normalized collision energy of 40%.

### Peptide quantification and identification by MaxQuant and Mascot

Raw MS files were processed using the MaxQuant software (ver. 1.0.14.7, Max-Planck Institute of Biochemistry, Department of Proteomics and Signal Transduction, Munich) by which the precursor MS signal intensities were determined and HCD-MS/MS spectra were de-isotoped and filtered such that only the ten most abundant fragments per 100-*m*/*z* range were retained. Peptides were identified by searching all MS/MS spectra against a concatenated forward/reversed target/decoy version of a mouse IPI v.3.37 protein sequence database including protein sequences of common observed contaminants like human keratins and porcine trypsin. The HCD-MS/MS spectra were searched with variable modifications of oxidation (M), acetylation (prot. N-term), Gln->pyro-Glu, Amidation (C-term), phospho (STY) and no enzyme specificity required. Search parameters were set to an initial precursor ion tolerance of 7 p.p.m. and MS/MS tolerance at 0.02 Da. Label-free peptide quantification based on extracted ion chromatograms and spectral counts and validation was performed in the MaxQuant software suite^35^ requiring a minimum Mascot score of 10 at a fixed peptide false discovery rate (FDR) threshold of maximum 2.5% and a fixed protein FDR threshold of maximum 1.5% to achieve a final peptide FDR<0.01. Phosphorylation sites were considered localized at a localization probability above 75%. The minimum required peptide length was set to six amino acids and peptide precursors were filtered on individual peptide mass errors after nonlinear post-acquisition recalibration^36^. The mass spectrometry proteomics data have been deposited to the ProteomeXchange Consortium via the PRIDE partner repository with the dataset identifier PXD002431.

### Data analysis

The combined analysis of protein orthologous groups from different species annotated with information from several databases required programming to integrate all layers of information. Here, this was all done in the programming language Perl together with freely available tools for blast and alignment. References are provided below and a simplified script is available as Supplementary Data 6 with instructions for use in Supplementary Note 1 and license information in Supplementary Note 2.

### Mammalian orthologous protein groups and mapping scheme

This used the existing non-supervised orthologous protein groups for mammals (maNOGs), which was downloaded from eggNOG 3.0 (http://eggnog.embl.de/version_3.0/). The sequence database was downloaded from STRING 9.0.5 (http://string-db.org) together with the alias files for the STRING proteins (http://string-db.org/). Everything was filtered to only contain rat, mouse and human species identifiers. The Uniprot/Swissprot (http://uniprot.org) sequences for rat, mouse and human species and the IPI sequences for rat and mouse were downloaded (ftp://ftp.ebi.ac.uk/pub/databases/IPI). Mapping of these databases to the STRING database was done using blastp (http://blast.ncbi.nlm.nih.gov/) and the alias protein file was supplemented with these protein mappings. This created the sequence mapping framework for the maNOGs.

### Annotation of orthologous protein groups

To annotate the maNOG framework, information from three different sources were integrated:

- Proteins in Uniprot (http://uniprot.org) that contain the sequence feature annotation ‘Peptide' indicates this part of the protein contains ‘active peptides processed from a larger precursor protein and with a well-defined biological activity'. These parts annotated as ‘Peptide' was extracted and mapped to the maNOG identifier for the species rat, mouse and human.
- The Neuropeptide Database (www.neuropeptides.nl) contains a list of gene names and precursor names. These were extracted and mapped to the maNOG identifier.
- The Sweden peptide Database (www.swepep.org) was acquired and the annotated peptides were mapped to the maNOG identifiers through the Uniprot identifier information.

The maNOGs through which any of these entries received an annotation was marked as a ‘pro-hormone precursor' and the peptide was stored in a flatline format for later retrieval.

### Annotation of identified peptides and proteins

Through the mapping scheme described above it was now straightforward to add a level of maNOG information to the list of protein groups identified from the mass spectrometry MaxQuant analysis. Any information on ‘pro-hormone precursor' was included in this annotation. A complication was that the identification list describes protein groups in contrast to single proteins. Proteins in such a protein group are indistinguishable based on the observed peptides and was rarely found to map to different maNOGs. In the cases where a single maNOG uniquely could explain all peptides, this identifier was kept. Alternatively, if only multiple maNOGs could explain the peptides, a group of maNOGs were formed hereby extending the positive annotation from a single maNOG to all maNOGs in the maNOG group.

### Sequence alignment

The protein sequences present within a maNOG group were aligned using the software tool Muscle 3.8 (ref. 37). All shorter peptides were subsequently mapped onto this alignment.

### Assembly of LPV

Identified peptides belonging to the same maNOG group were found to show a large degree of overlap. To reduce this, all overlapping peptides were iteratively merged to a longer sequence based on the largest overlap between any pair of sequences. In detail, the algorithm was the following:

- Define a unique set of peptides mapping to a maNOG entry or group.
- Define overlap in sequences as the number of shared amino acids in peptide sequences with no gaps allowed. It is allowed for one sequence to extend in N-terminal and/or one sequence to extend in the C-terminal direction. A minimum length of overlap was set to two amino acids.
- Define a combined peptide sequence as two sequences merged to form the shortest sequence that contains them both while checking that the combined sequence is a part of one of the protein sequences in the maNOG.
- Define site-specific modifications as blockers of overlap past the modified residue. Only modifications of possible *in vivo* origin were considered: N-terminal acetylation, pyro-glutamic acid and amidation of C-terminal.
- Calculate the number of overlap in amino acids between all pair of peptide sequences based on i-iv.
- Replace the pair with the largest allowed with the combined sequence.
- The two previous points are repeated iteratively until as much as possible is combined and no overlapping sequences are found.
- The final list of peptides constitutes the minimum set that can explain all other peptides and is referred to as the LPV.

### Calculation of occupancy

A moving window approach where the quantitative measure for each amino acid is based on how many spectra has been identified containing this amino acid. This is a form of spectral count on amino-acid level. For phosphorylation, site specificity was kept separate from quantification. This means that all possible phosphorylation sites were from a quantitative perspective considered to be phosphorylated in all peptide sequences that were found phosphorylated. The occupancy was then calculated by dividing the amino-acid spectral count for only phosphorylated peptides compared with an amino-acid count for the matching amino acid when all peptide sequences were included.

### Logo plots

Amino-acid sequence logo plots were created using IceLogo 1.2 (ref. 38). For the N- and C-terminal plots, the three up- or downstream amino acids were read in for each peptide. The same was done for LPVs or amidated LPVs. In the phosphorylation site plots, the alignment was performed based on the phosphorylated site. For all logo plots, scaling in IceLogo was done based on the amino-acid frequencies found in *Mus musculus*.

### Visualization file

For peptides mapping to pro-hormone precursors, visualization was done as follows. Protein sequences were downloaded from Uniprot and Ensembl for rat, mouse and human with their accession identifier and file origin kept in a hyperlink. Sequences were aligned within each group as explained above. An in-house Perl script formatted this, where one line was a sequence. Onto this alignment, the identified sequences were mapped first followed by the LPVs and finally by the known reference peptides from the annotation effort performed under ‘Annotation of Orthologous Groups'. Modifications for possible *in vivo* origin, namely pyro-Gln, amidation, acetylation and phosphorylation were included by adding a parenthesis and a modification name. To ease interpretation, everything was exported to Excel, where each entry was a sheet and where colouring was applied to differentiate the different type of sequences and highlight modifications. Hyperlinks were used to link to origin of sequences. The visualization result can be found in Supplementary Data 3.

### GO analysis

GO enrichment analysis of the peptidome data set was performed using the human orthologues of protein entries identified by at least two peptides and compared with the filter-retained proteome as background with the innateDB software tool (http://www.innatedb.ca). Enriched pathways and GO-terms for molecular function and cellular component were determined based on their corrected *P*-values, requiring at least ten input genes in a group. *P*-values were calculated using a hypergeometric test and corrected for multiple testing with a Benjamini–Hochberg. A cutoff of *P*<0.01 was applied and overrepresented terms visualized as bars in Fig. 1d, where the length is calculated as -10*log10(P).

### MC receptor assays

MC-binding assays were performed as radioligand displacement filtration binding assays to membranes from recombinant BHK570 cells expressing the relevant human melanocortin receptor using ^125^I-NDP-α-MSH as radio ligand^26^. The IC50 values were calculated by nonlinear regression analysis of binding curves (six data points minimum) using the programme GraphPad Prism (GraphPad Software). Ki values were calculated from IC50 according to the Cheng−Prusoff equation. Each assay was performed in duplicate. Data points are average values from the assays and s.e.m. values are indicated with error bars (*n*=3–9).

The functional activities of the analogues on the MC receptors were studied in intact BHK cells expressing the human MC receptors by measuring the cAMP production after stimulation with the analogues. The assay was performed in BHK570 cells expressing the MC receptors, which were stimulated with increasing concentrations of potential MC receptor agonists in a range of 10^−11^ to 10^−5^ M, and the degree of stimulation of cAMP was measured using the Flash Plate cAMP assay kit (NEN Life Science Products cat no SMP004) using the supplied materials and buffers^26^. The assay was performed in duplicates and each compound was tested six times. EC50 values were calculated by nonlinear regression analysis of dose response curves (six data points minimum) using GraphPad Prism (GraphPad Software; EC50 of α-MSH and phosphorylated α-MSH were 25 nM (*n*=7) and 251 nM (*n*=10)).

For statistical analysis, unpaired two-tailed *t*-test were used to compare the pEC50 values and unpaired *t*-test with Welsch correction were used to compare the Emax values.

### Synthesis of phospho α-MSH

Peptides were synthesized using standard Fmoc chemistry. Phospho-serine was introduced by using Fmoc-Ser(PO-(OBzl)OH)-OH (Novabiochem). The peptide was cleaved from resin with 95% TFA, 2% triisopropylsilane (TIPS) and 2.5% water, for 2 h and isolated by precipitation with ether. The crude peptides were purified on a reverse phase preparative HPLC using a C18 column (Waters XBridge PrepC18, 5 μm, 50 × 250 mm^2^), with an acetonitrile/water 0.1% TFA gradient from 8 to 28% over 40 min. Fractions containing the pure peptide were collected and lyophilized.

## Additional information

**Accession codes**: The mass spectrometry proteomics data have been deposited to the ProteomeXchange Consortium via the PRIDE partner repository^36^ with the dataset identifier PXD002431.

**How to cite this article**: Secher, A. *et al*. Analytic framework for peptidomics applied to large-scale neuropeptide identification. *Nat. Commun.* 7:11436 doi: 10.1038/ncomms11436 (2016).

## Supplementary Material

Supplementary Information Supplementary Figures 1-2 and Supplementary Notes 1-2

Supplementary Dataset 1 All identified peptides. List of 16,037 unique modification-specific peptide variants identified by LC-MS/MS covering 14,416 unique peptide sequences.

Supplementary Dataset 2 Longest peptides after grouping. List of 2,835 LPVs reduced from the 14,416 unique peptides. Of which 356 LPVs belonged to the pro-hormone precursor group.

Supplementary Dataset 3 Manog visualized neuropeptide database. The full list of peptides and the resulting LPVs aligned against their pro-hormone precursor compiled in a database format for easy visualization and analysis.

Supplementary Dataset 4 Potential new neuropeptides. List of potential, biologically active neuropeptides generated from the LPVs.

Supplementary Dataset 5 Phosphorylation sites. List of localized phosphorylation sites identified.

Supplementary Dataset 6 Simplified script. Perl script to create the Longest Peptide Variants (LPVs) from a list of peptide sequences.

## Figures and Tables

**Table 1:** Extract of potential new neuropeptides.

maNOG description	Sequence (N-terminal. Peptide sequence amino acids. C-terminal)
Chromogranin-A	EKR.LEGEDDPDRSMKLSFRARAYGFRDPGPQL.RRG
Chromogranin-A	NRR.AEDQELESLSAIEAELEKVAHQLQALRR*.G
Cocaine- and amphetamine-regulated transcript protein	PRR.QLRAPGAVLQIEALQEVLKKLKS.KRI
Corticotropin-releasing hormone	AER.GAEDALGGHQ*.GAL
Galanin	EKR.GWTLNSAGYLLGPHAIDNHRSFSDKHGLTG.KRE
Galanin	GKR.ELPLEVEEGRL*.GSV
Glucagon	DKR.HSQGTFTSDYSKYLDS.RRA
Glucagon	GRR.DFPEEVAIAEEL*.GRR
Kisspeptin-1	VQR.EKDMSAYNWNSFGLRY*.GRR
Neuropeptide S	MKR.SFRNGVGSGVKKTSF.RRA
Neurosecretory protein VGF	ATR.QAAAQEERLADLASDLLLQYLLQGGARQRDLG*.GRG
Neurosecretory protein VGF	VRR.LEGSFLGGSEAGERLLQQGLAQVEAG.RRQ
Nucleobindin-2	EKR.KEEEAKFAEM.KRK
Pituitary adenylate cyclase-activating polypeptide	TKR.HSDGIFTDSYSRY.RKQ
Pituitary adenylate cyclase-activating polypeptide	YRK.QMAVKKYLAAVL*.GKR
Proenkephalin-A	MKK.DADEGDTLANSSDLLKELLGTGDNRAKDSHQQESTNNDEDSTSKRYGGFMRGL.KRS
Proenkephalin-A	MKR.YGGFMKKMDELYPVEPEEEANGGEILAKRYGGFM.KKD
Proenkephalin-A	QKR.YGGFMRRV*.GRP
Proenkephalin-A	QKR.YGGFMRRVGRPEWWMDYQKRYGGFL.KRF
Pro-FMRFamide-related neuropeptide FF	FGR.NAWGPWSKEQLSPQAREFWSLAAPQRF*.GKK
Pro-FMRFamide-related neuropeptide VF	SPR.ARANMEAGTMSHFPSLPQRF*.GRT
Progonadoliberin-1	DLR.GALERLIEEEA*.GQK
Prohormone convertase 2	HKR.QLERDPRIKMALQQEGFD.RKK
Prohormone convertase 2	SKR.NQLHDEVHQW.RRN
Pro-opiomelanocortin	FKR.ELEGEQPDGLEHVLEPDTEKADGPYRVEHFRWGNPPKD.KRY
Pro-opiomelanocortin	GKK.RRPVKVYPNVAENES†AEAFPLEF.KRE
Pro-opiomelanocortin	GKR.SYS†MEHFRWGKPVGKK.RRP
ProSAAS	LRR.AVDQDLGPEVPPENVL*.GAL
Protachykinin-1	GKR.DAGHGQISHKMAYERSAMQNYE.RRR
Protachykinin-1	GKR.DAGHGQISHKRHKTDSFVGLM*.GKR
Protachykinin-1	HKR.HKTDSFVGLMG.KRA
Pro-thyrotropin-releasing hormone	ERR.FLWKDLQRVR*.GDL
Pro-thyrotropin-releasing hormone	GKR.EEEEKDIEAEER*.GDL
Pro-thyrotropin-releasing hormone	GKR.EEEEKDIEAEERGDLGEGGAWRLH.KRQ
Pro-thyrotropin-releasing hormone	TKR.QHPGRRFIDPELQRS†WEEKEGEGVLMPE.KRQ
Pro-thyrotropin-releasing hormone	VKR.QHPGRRSFPWMESDVT.KRQ
Secretogranin-1	EKR.KRLGALFNPYFDPLQWKNSDFE.KKG
Secretogranin-1	EKR.PFSEDVNW*.GYE
Secretogranin-1	EKR.SFARAPHLDL.KRQ
Secretogranin-1	LRK.SGKEVKGEEKGENENSKFEVRLLRDPSDASV*.GRW
Secretogranin-1	NKR.SEASAKKKEESVARAEAHFVELEKTHS†REQSSQESGEET.RRQ
Secretogranin-1	TRR.QEKPQELPDQDQSEEES†EEGEEGEEGATSEVT.KRR
Secretogranin-1	YKR.NHPDSELESTANRHS†EETEEERSYEGAKGRQHRGRGREPGAYPALDS†RQE.KRL
Secretogranin-2	LKR.VPSPGSSEDDLQEEEQLEQAIKEHL*.GQG
Secretogranin-2	LKR.VPSPGS†SEDDLQEEEQLEQAIKEHLGQGSS†QEMEKLAKVS.KRI
Secretogranin-2	MKR.SGHLGLPDE*.GNR
Secretogranin-2	SKR.IPAGSLKNEDTPNRQYLDEDMLLKVLEYLNQEQAEQ*.GRE
Secretogranin-2	SKR.IPAGSLKNEDTPNRQYLDEDMLLKVLEYLNQEQAEQGREHLA.KRA
Secretogranin-5	RKR.RSVNPYLQ*.GKR
Tachykinin-3	QKR.DMHDFFVGLMG.KRN
Urocortin 3	SKK.NFGYLPTQDPS†GEEEDEQKHIKN.KRT
Urocortin 3	VKK.NKLEDVPVLS.KKN
VIP peptides	GKR.ISSSISEDPVPV.KRH
VIP peptides	LRK.QMAVKKYLNSILN*.GKR

^*^Indicates amidated C-terminal.

^†^Indicates phosphorylated amino acid.
