# Supplementary material for: Analytic framework for peptidomics applied to large-scale neuropeptide identification
Source: Nat Commun. 2016 May 4;7:11436. doi: 10.1038/ncomms11436 (PMC4857386; doi:10.1038/ncomms11436)
Supplement: Supplementary Information — Supplementary Figures 1-2 and Supplementary Notes 1-2 [file ncomms11436-s1.pdf]

Supplementary Figure 1: MS Data Quality (1/2)

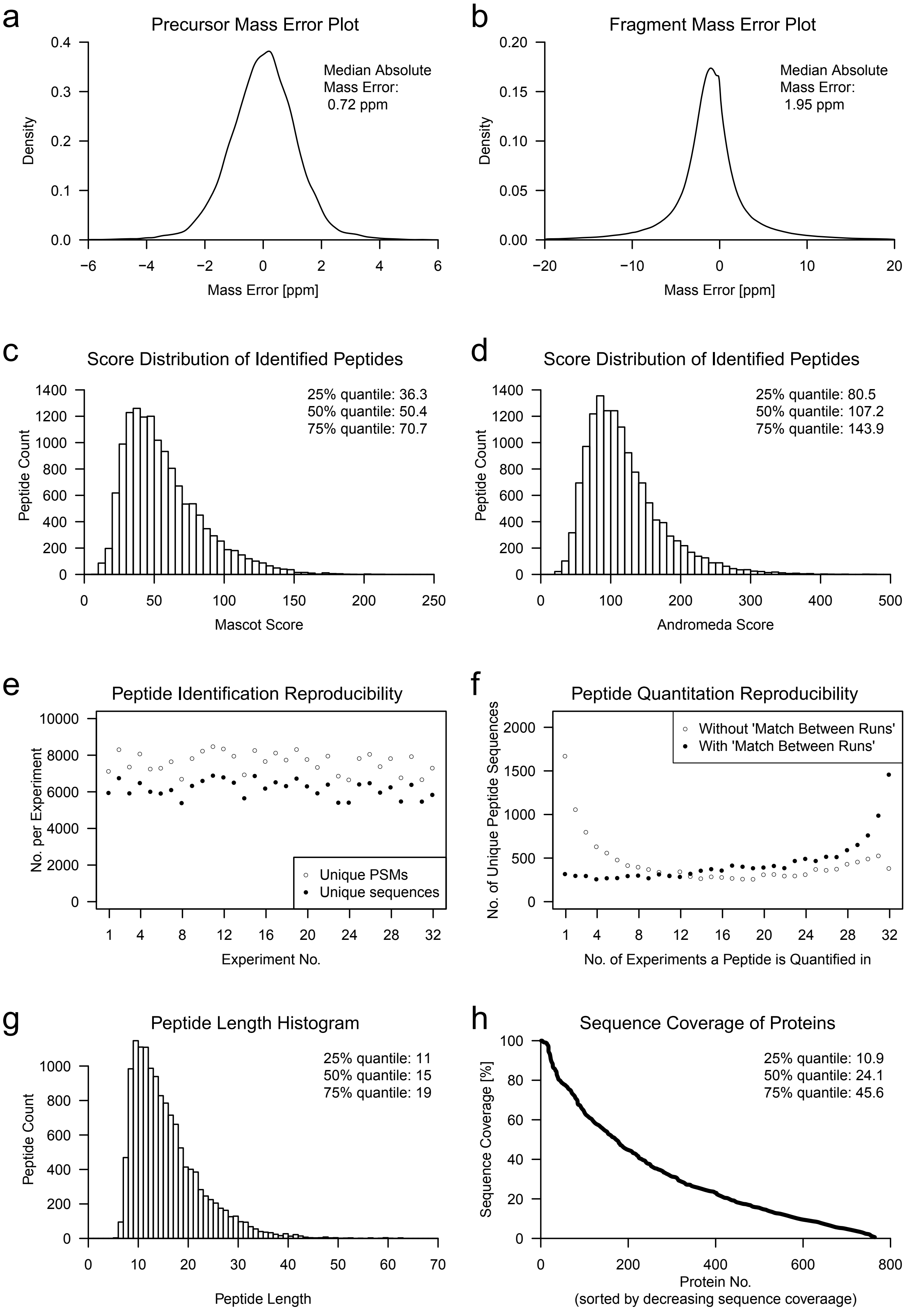

Supplementary Figure 1: MS Data Quality (2/2)

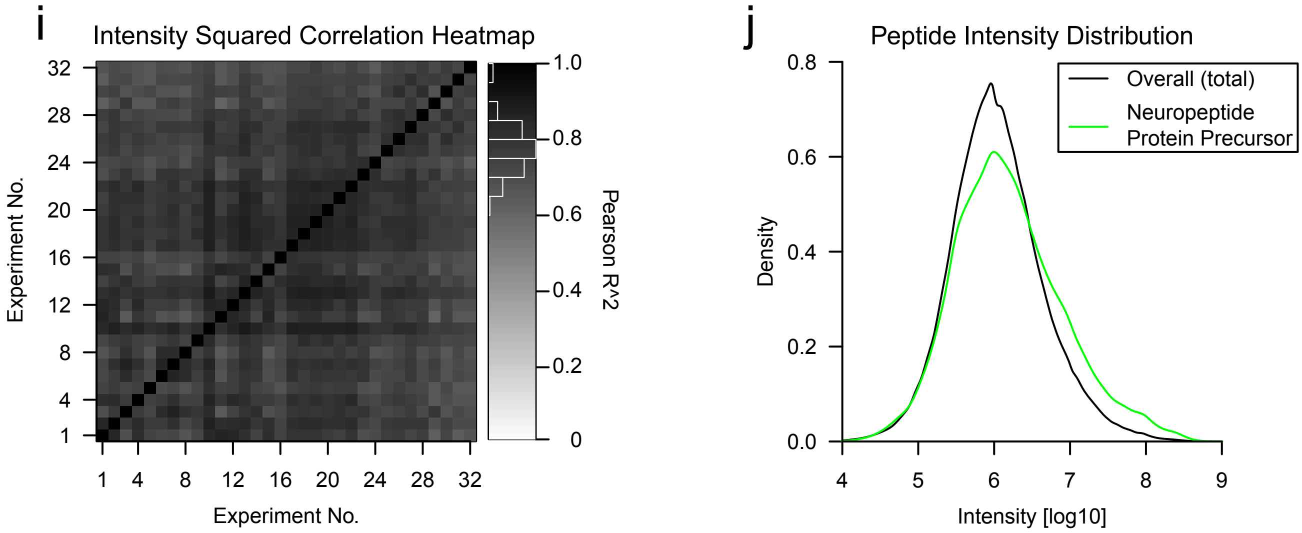

Supplementary Figure 1: (a) Kernel density estimation plot of all precursor mass errors for all identifications. (b) Kernel density estimation plot of all fragment mass errors for fragment ion assignments. (c) Mascot score distribution of all identifications. (d) Andromeda score distribution for all identifications. (e) Scatter plot of the number of unique PSMs and peptide sequences observed across 32 replica. (f) Scatter plot of the number of unique peptide sequences versus how many experiments a peptide is observed in. The match between run feature enables a greater overlap between experiments. (g) A histogram of the length distribution of the unique peptide sequences. (h) Peptides mapped to proteins and the observed sequence coverage. (i) Heatmap comparison of all quantification events across all samples with pairwise complete squared pearson correlation visualized on a grey-scale. (j) Kernel density estimations for peptides either all identifications or identifications mapping to neuropeptide protein precursors (the area under curve are identical).

Supplementary Figure 2:
Comparison with Prediction Tools

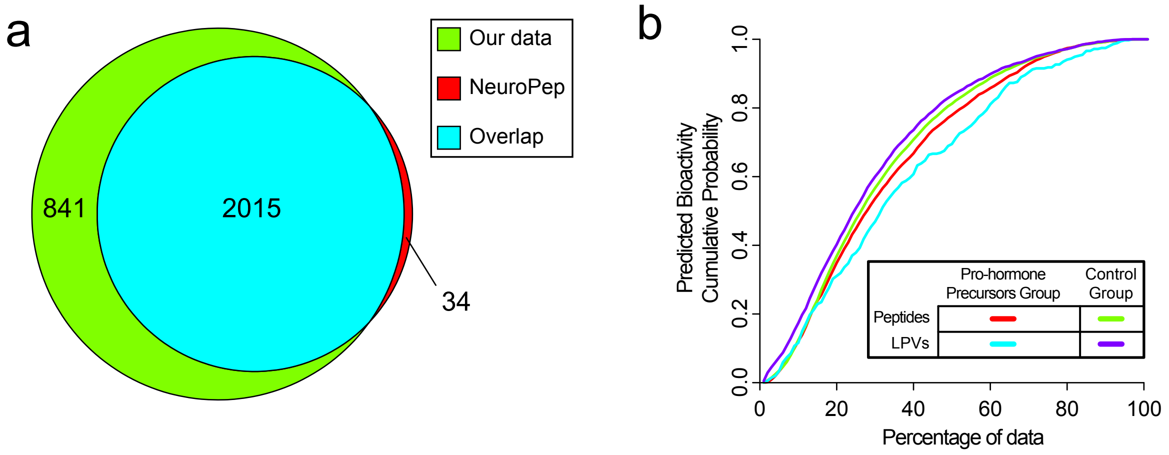

a) Venn diagram showing peptides overlap between pro-hormone precursors and sequences present in NeuroPep. b) Cumulative probability plot for predictions from PepRanker on bioactivity for peptides and LPVs coming from pro-hormone precursors or not where 1 indicates high probability for bioactivity.

Supplementary Note 1:
Software Instructions

Instructions for use of the script: annotatePeptides.pl

The script is written in Perl and requires a Perl interpreter installed. It is usually part of the base installation of most Linux, Unix, and MacOS but Microsoft Windows requires installation of seperate software. This could be ActivePerl (http://www.activestate.com/activeperl/downloads) or Strawberry Perl (http://strawberryperl.com/). Please check license demands before download.

The script expects the following files to be present in the same directory as the script:
- "peptideList_Example.txt", a text file containing the peptides from a shotgun experiment.
- "combined_fasta.txt", this file contains "positive" proteins that the peptides are assembled against.

The script is executed either by simple clicking or by command line:
perl annotatePeptides.pl

The output file (default: "annotatedPeptideList.txt") contains three columns, the first being the peptide sequence, the second the ID of the protein it matches to and third being the Longest Peptide Variant (LPV) of which this peptide sequence is associated with.

Supplementary Note 2:
Software License

Copyright (c) 2016 University of Copenhagen, Denmark

Permission is hereby granted, free of charge, to any person obtaining a copy of this software and associated documentation files (the "Software"), to deal in the Software without restriction, including without limitation the rights to use, copy, modify, merge, publish, distribute, sublicense, and/or sell copies of the Software, and to permit persons to whom the Software is furnished to do so, subject to the following conditions:

The above copyright notice and this permission notice shall be included in all copies or substantial portions of the Software.

THE SOFTWARE IS PROVIDED "AS IS", WITHOUT WARRANTY OF ANY KIND, EXPRESS OR IMPLIED, INCLUDING BUT NOT LIMITED TO THE WARRANTIES OF MERCHANTABILITY, FITNESS FOR A PARTICULAR PURPOSE AND NONINFRINGEMENT. IN NO EVENT SHALL THE AUTHORS OR COPYRIGHT HOLDERS BE LIABLE FOR ANY CLAIM, DAMAGES OR OTHER LIABILITY, WHETHER IN AN ACTION OF CONTRACT, TORT OR OTHERWISE, ARISING FROM, OUT OF OR IN CONNECTION WITH THE SOFTWARE OR THE USE OR OTHER DEALINGS IN THE SOFTWARE.
